# LBS-YOLO: a lightweight model for strawberry ripeness detection

**DOI:** 10.3389/fpls.2025.1715263

**Published:** 2025-12-04

**Authors:** Haitao Fu, Xueying Li, Zheng Li, Li Zhu, Yuxuan Feng

**Affiliations:** College of Information Technology, Jilin Agricultural University, Changchun, China

**Keywords:** strawberry, YOLOv11, object detection, ripeness, lightweight

## Abstract

**Introduction:**

The traditional strawberry picking operation has long relied on manual work. With the aging trend of the population becoming more and more obvious, the application of intelligent picking technology has become an irreversible trend. However, existing recognition methods still face bottlenecks such as suboptimal recognition accuracy and low computational efficiency. To address these issues, this study constructs a lightweight detection model, LBS-YOLO, based on an improved YOLOv11n architecture, significantly the model’s accuracy and interference robustness while greatly compressing the parameter quantity.

**Methods:**

The LBS-YOLO model is built upon YOLOv11n as the baseline network. In order to enhance the ability of backbone network feature representation, the model designs a lightweight LAWDS module. This design combines channel attention with spatial reconstruction operation to optimize the information retention efficiency in the down-sampling process, thus effectively enhancing the multi-scale feature representation ability and gradient flow propagation performance. Then in the feature fusion stage, the model introduces a Bidirectional Feature Pyramid Network (BiFPN), which not only enables cross-scale feature fusion but also achieves adaptive weighting through a learnable weight allocation mechanism. At last, adopts the C3k2_Star module to replace the conventional C3K2 for improved feature representation.

**Results:**

On the used strawberry dataset, the LBS-YOLO model reached 88.6% mAP@0.5 and 75.8% mAP@0.5:0.95, which were 2.2 and 1.3 percentage points higher than YOLOv11n, respectively. The LBS-YOLO model improves the recall rate from 83.2% of YOLOv11n to 86.4%, and the F1-score from 81.2% to 82.9%. Its computational complexity is 6.6 GFLOPs and its reasoning speed is 260.7 FPS. Even better, LBS-YOLO only needs 3.4MB of storage space and 1.6 million parameters, which are 34.6% and 38% less than YOLOv11n respectively.

**Discussion:**

The experiment demonstrates that, the LBS-YOLO model can significantly reduce the number of parameters and effectively improve the detection accuracy and operation efficiency. It successfully alleviated the problems of false detection and missed detection, thereby providing reliable technical support for strawberry growth monitoring, maturity identification and automatic picking.

## 1 Introduction

Strawberries belong to the Rosaceae family and are a fruit with low calorie content and extremely high water content, often recommended for weight-loss diets and in combating obesity (Hernández-Martínez et al., 2023). They are rich in various essential minerals and vitamins, and regular consumption helps improve blood lipid profiles and inflammatory markers, promotes cardiovascular health, exerts positive regulatory effects on bones, the brain, and gut microbiota, making them highly favored by consumers of all age groups (Newerli-Guz et al., 2023; Charoenwoodhipong et al., 2025). With the continuous advancement of facility cultivation technology, strawberries can be supplied continuously during non-traditional seasons such as winter and early spring, significantly extending the market availability period (Hodgdon et al., 2024). Off-season strawberry often commands higher market prices due to limited supply and strong market demand, thereby substantially increasing Planting income (Adams et al., 2021). However, Strawberry is a Finely planted crop, and the fruit firmness decreases sharply upon ripening, Delayed harvesting leads to over-ripening, rotting, and deterioration of the fruit, promoting the proliferation of large amounts of Harmful bacteria and significantly increasing the Risk of disease occurrence and spread within the greenhouse (Tenea and Reyes, 2024). Moreover, it is necessary to harvest strawberries in batches, which can not only ensure that strawberries are picked at the best maturity, but also enable strawberries to get on the market first, get higher prices, and improve income and people’s demand for strawberries (Zeneli et al., 2024).

Traditional strawberry harvesting operations are highly dependent on manual labor and commonly face issues such as low harvesting efficiency and an increasingly scarce workforce (Pinochet and Agehara, 2024). In recent years, deep learning technology has achieved significant progress in the realm of intelligent strawberry harvesting and has become a core driving force for automating and precisely managing fruit and vegetable harvesting operations (Guo et al., 2024). Zhou et al. proposed an improved Faster-RCNN optimized via transfer learning, replacing the fully connected layer and classification layer with a three-layer adaptive network and adding a dropout layer to reduce overfitting. The strawberry extraction accuracy exceeded 86%, with an overall accuracy of approximately 85% (Zhou et al., 2020). Zhang Xiaohua et al. proposed a method based on EfficientDet-D1, using EfficientNet as the backbone and optimizing compound scaling coefficients. The strawberry detection mAP reached 97.50%, with an average detection time of 0.34s, meeting the requirements for rapid strawberry detection and classification (Zhang et al., 2022). Li et al. improved the Faster R-CNN model by replacing RoiPooling with RoiAlign to eliminate quantization errors and adopted bilinear interpolation to retain floating-point values, thereby reducing model error, the mAP reached 0.8733, with counting accuracies of 99.1% for mature strawberries and 73.7% for immature strawberries (Li et al., 2023). Li et al. proposed the lightweight SGSNet, constructed a full growth cycle strawberry dataset, and adopted GrowthNet as the backbone, incorporating DySample for adaptive upsampling and an iRMB-optimized feature fusion module. The model achieved a recall of 99.45%, mAP@0.5 of 99.50%, with 5.86 million parameters and a computational cost of 14.7 GFLOPs (Li et al., 2024).

Presently, the application of YOLO series models in strawberry detection and recognition tasks has continuously deepened (Balaji et al., 2025). With multiple studies advancing model performance through Lightweight design, fusion of attention mechanisms, and optimization of feature extraction modules. Yu et al. proposed the Ripe-Detection framework, which stands out in balancing Lightweight design with high accuracy, achieving an mAP50 of 96.4% and a 46% decrease in computation (Yu et al., 2025). Liu et al. introduced a remix channel-spatial attention mechanism that simultaneously focuses on key features along the channel dimension and the Strawberry regions in the spatial dimension, reducing background interference, the constructed YOLOv11-HRS model shows an elevation of 3.4% in mAP@0.5, with a 19% reduction in Number of parameters (Liu et al., 2025). Tao et al. based on a public dataset and a slipstream dataset supplementing special scenes, proposed the YOLOv5s-BiCE algorithm, adopting a Biformer dual-attention mechanism to highlight Strawberry target traits and suppress irrelevant info across both channel and spatial dimensions, and this approach improves the mean average precision and accuracy by 2.8% and 7.4%, respectively, further enhancing detection accuracy on top of the Lightweight model (Tao et al., 2024). Yang et al. proposed the LS-YOLOv8s model, which captures long-range feature dependencies through window attention to effectively handle the discontinuity of strawberry features under leaf occlusion, while improving the residual structure to enhance feature transmission efficiency and reduce feature loss in complex environments, and on the validation set, the model demonstrates significantly superior performance compared to existing state-of-the-art approaches, achieving an improvement in mAP0.5 (Yang et al., 2023).

Numerous studies also focus on robustness and small object detection performance in complex environments, and significant progress has been achieved in the collaborative optimization of precision and efficiency. He et al. proposed the RLK-YOLOv8 model, which effectively enhanced the model’s feature extraction capability and multi-scale target detection performance in complex occluded environments by introducing modules such as the large-kernel convolution RepLKNet and dynamic head DynamicHead, ultimately achieving a 95.4% mAP (He et al., 2025). Gao et al. constructed the WCS-YOLOv8s model, which significantly improved the model’s recognition robustness and generalization performance in small target scenarios such as the flowering and fruiting period by adopting Warmup data augmentation and the SE-MSDWA attention module (Gao et al., 2025). Ma et al. proposed the YOLOv11-GSF model, utilizing GhostConv lightweight convolution and the F-PIoUv2 loss function, which ensured high accuracy with an AP@50 of 97.8% while significantly reducing computational overhead (Ma et al., 2025). Wang et al. proposed a YOLOv8+ model integrating ECA attention and Focal-EIOU loss, combined with image processing-based red proportion analysis of the centerline, achieving a detection accuracy of 97.8% and a maturity classification accuracy of 91.9%, while maintaining high speed, thus enabling robust detection of small strawberry targets and maturity grading under complex conditions (Wang et al., 2024).

In the research of the existing strawberry recognition model above, the performance of the model has been continuously improved through various methods such as module reorganization, attention mechanism introduction and loss function optimization. At present, although the improvement of the algorithm has made obvious progress, it still faces some challenges in practical application: there is no basis for strawberry maturity grading, which is different from the agricultural industry standard, and it is not suitable for fine operation requirements, branches and leaves, fruit occlusion and other factors are easy to cause misjudgment and missed detection, which affects the reliability of the system. At the same time, the balance between accuracy and efficiency has not been well solved.

Therefore, in order to solve the above problems. In this study, the YOLOv11n model has been improved, and its innovation is mainly reflected in the following aspects:

1. According to the agricultural industry standard data set, the original maturity level is subdivided into “suitable for transportation and storage” and “suitable for local sales”, and the harvested strawberries do not need to be manually classified again.
2. Lightweight Adaptive Weighted Downsampling Module (LAWDS) is designed and applied in the research. It uses attention mechanism to adaptively retain key features, which significantly reduces information loss.
3. A C3k2_Star module integrating depthwise separable convolution with a gating mechanism was constructed, significantly enhancing the network’s feature discrimination capability. Introduced the Bidirectional Feature Pyramid Network (BiFPN), enabling adaptive optimization of multi-scale feature fusion.

These improvements allow the proposed LBS-YOLO model to accurately identify key growth stages of strawberries, providing more effective technical support for the realization of intelligent strawberry harvesting.

## 2 Materials and methods

### 2.1 Strawberry dataset construction

#### 2.1.1 Dataset source and classification

**Figure 1:**
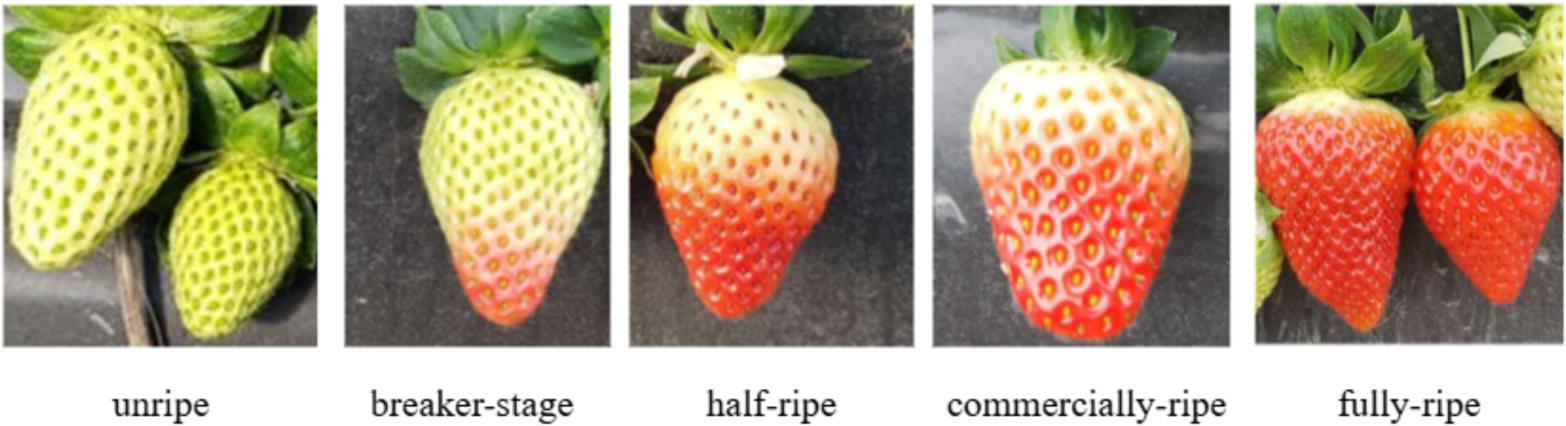
Examples of strawberries at different maturity levels.

The dataset used in this study is a publicly available dataset from Baidu Paddle. Detailed dataset information can be found at: https://aistudio.baidu.com/aistudio/datasetdetail/147119. A total of 3,000 strawberry images are included here. The original dataset categorizes strawberries according to different maturity stages into three specific classes: unripe, half-ripe, and ripe. In this study, based on the People’s Republic of China agricultural industry standard NY/T 2787-2015 (Yu et al., 2015), maturity is further subdivided into five categories. Unripe stage, characterized by entirely white or green color; breaker-stage, where the majority of the strawberry is green with small red areas appearing; half-ripe stage, where the red area increases to half or more of the entire strawberry surface; Commercially-ripe stage, where most of the strawberry is red, with coloring coverage reaching 80% or more. Fully-ripe stage, appearing entirely red or nearly entirely red, with coloring coverage reaching 90% or more. Each maturity level of strawberry is shown in **Figure 1**.

#### 2.1.2 Dataset annotation method

The GrabCut method is a tool for separating parts of an image. It uses graph cutting and Gaussian Mixture Models to work (Rother et al., 2004). By utilizing an initial bounding box provided by the user, this algorithm iteratively optimizes the color distribution models of foreground and background, and achieves pixel-level high-precision segmentation by minimizing an energy function. In this study, GrabCut algorithm was used to realize automatic extraction of strawberry target area, and complex background interference was effectively eliminated, thus laying a reliable foundation for subsequent maturity analysis.

**Figure 2:**
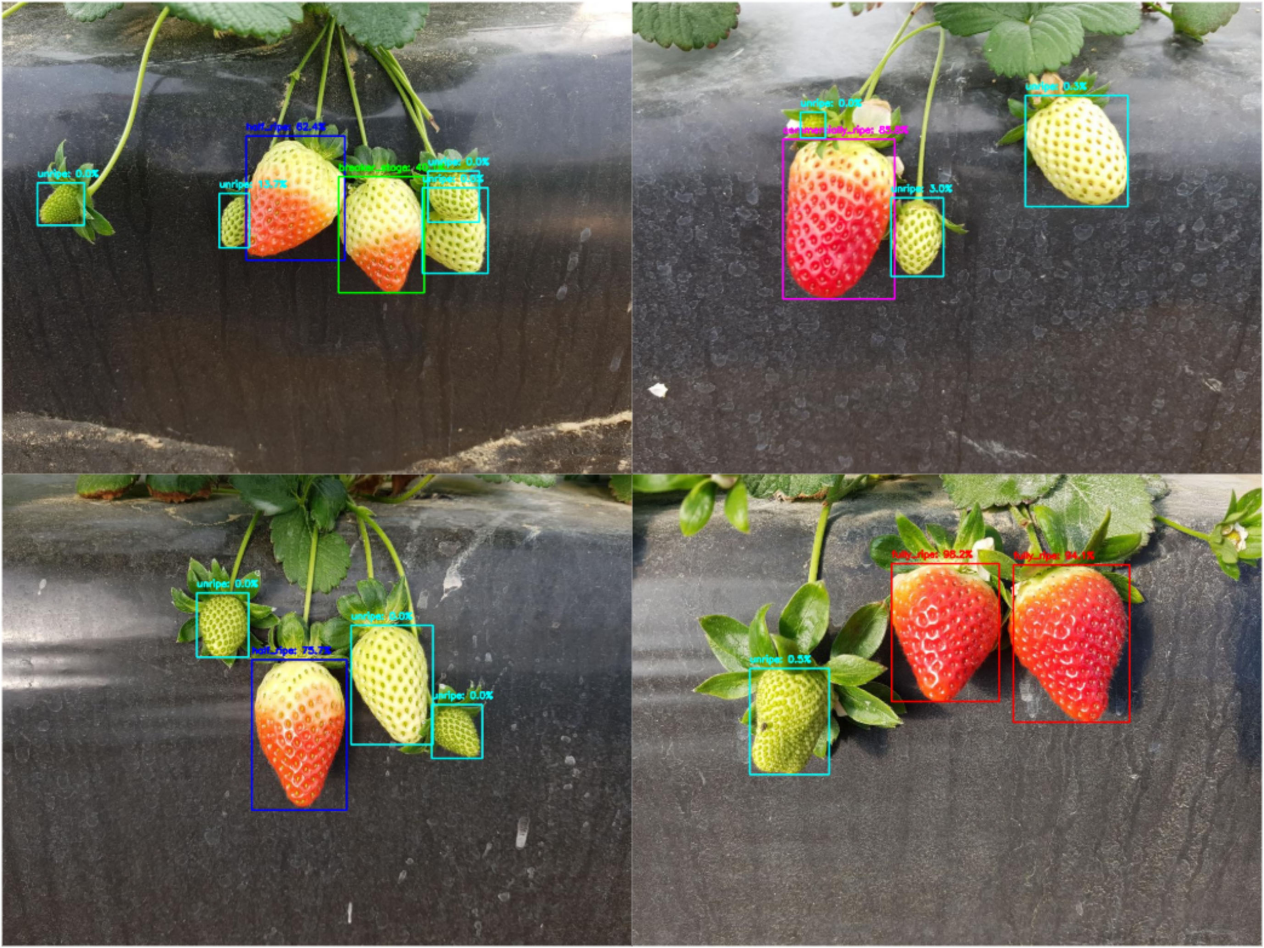
Example of maturity numerical calculation.

In order to reduce the error of artificial judgment on strawberry maturity and scientifically classify the maturity level, this study first uses GrabCut algorithm to segment the strawberry area and remove the background behind it, then converts the image into HSV color space, and then determines the maturity value according to the proportion of red pixels. Then, the professional labeling tool LabelImg is used to manually correct the samples with obviously misjudged or red areas caused by branches and leaves and fruits, and the maturity is underestimated. The calculation results of ripeness values are shown in **Figure 2**.

#### 2.1.3 Dataset splitting

We used the 3,100 public strawberry images and separated them for training, validation, and testing with a ratio of 7:2:1. Some categories in the dataset didn’t have enough samples, which could make the model perform poorly. To solve this and make the model stronger, we added more data specifically for these rare categories. The additional 1,000 augmented images were all incorporated into the training set, further optimizing data distribution and providing more balanced and standardized data support for model training.

### 2.2 Lightweight improved YOLOv11n model

In this study, based on the YOLOv11n model, the LAWDS module and C3k2_Star bottleneck unit were introduced into the backbone network, and the model volume was successfully reduced by 34.6% while maintaining the accuracy, and the multi-scale feature fusion was enhanced by using the BiFPN structure to improve the small target detection ability, and finally the number of detection head channels was optimized to 256. These improvements significantly enhance the performance of the model in strawberry maturity detection, and provide a more efficient solution for accurate maturity identification.

#### 2.2.1 Light adaptive-weight downsampling module

**Figure 3:**
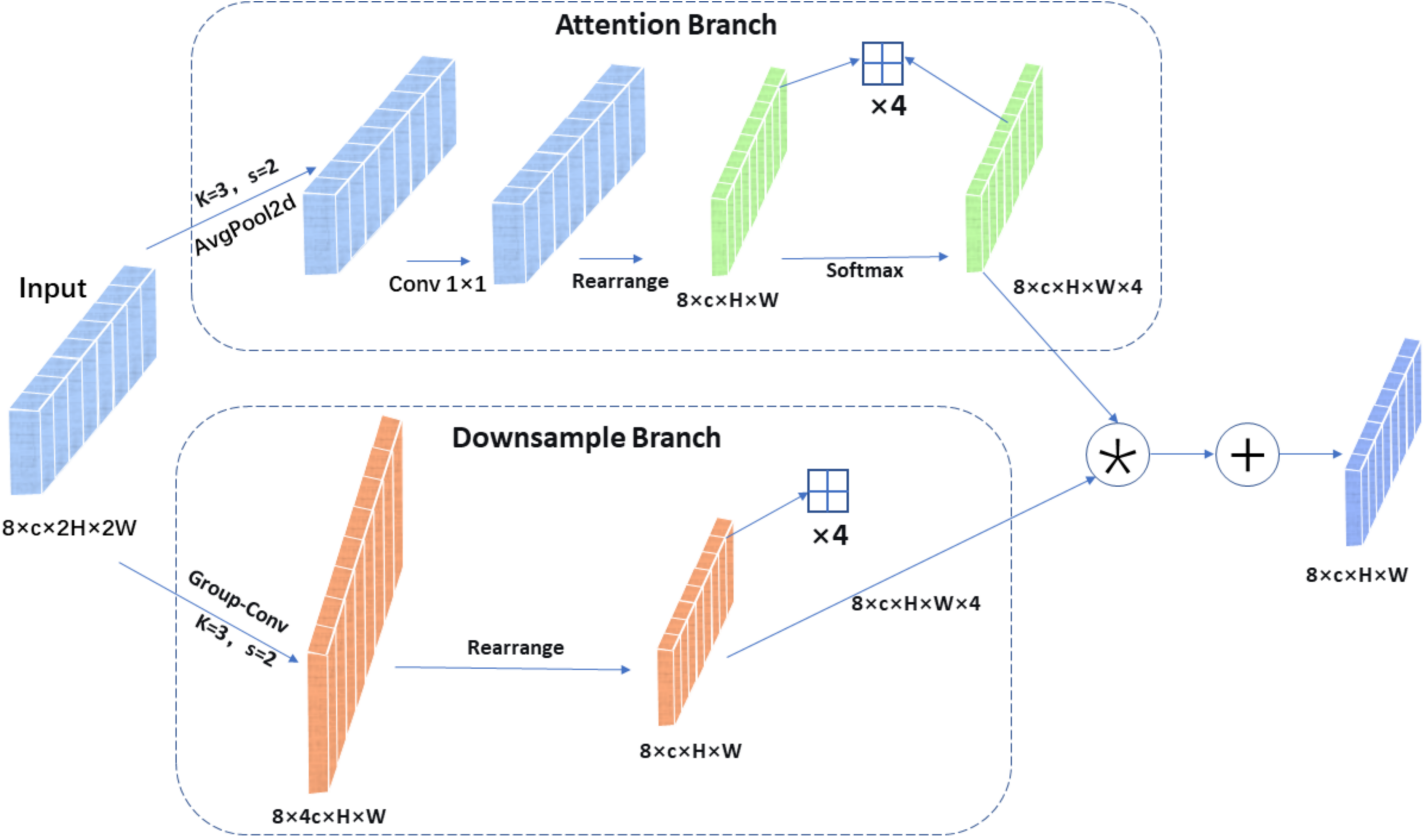
LAWDS module structure diagram.

A lightweight adaptive weighted downsampling (LAWDS) module is designed into the backbone network to alleviate the inherent information loss and aliasing effect of standard downsampling operation. The module first captures contextual information via average pooling and generates an attention weight map using a 1×1 convolution; subsequently, the weights are reshaped into a five-dimensional tensor and undergo channel expansion and downsampling through grouped convolution; finally, the features are adaptively fused by applying softmax-normalized weights to produce a feature map with half the spatial dimensions. Unlike methods employing fixed or static weights, LAWDS dynamically generates content-aware adaptive weights to guide the downsampling process, thereby prioritizing the preservation of salient features from the input and generating more informative feature representations even at reduced resolution. As shown in **Figure 3**.

Given an input feature tensor $\;X\in {\mathbb{R}}^{B\times C\times 2H\times 2W}$, where B is the batch size, C is the number of channels, and 2H×2W is the spatial dimension, the LAWDS module produces a downsampled output $Y\in {\mathbb{R}}^{B\times C\times H\times W}$. Its core operation is adaptive weighted fusion of features within local 2×2 windows. The computation process mainly consists of three stages:

Adaptive weight generation: A lightweight attention branch first processes the input to generate a spatial weight tensor A, which encodes the importance of each pixel within its local 2×2 context. As shown in Equation 1.

(1) $$\begin{array}{cc} A_{raw} & =f_{att}\left(X\right)=Conv_{1\times 1}\left(AvgPool_{3\times 3}\left(X\right)\right)\\ \;\;\;\;\;\;\;A & =Softmax\left(Rearrange\left(A_{raw}\right)\right) \end{array}$$

Here, ${\text{AvgPool}}_{3\times 3}\;$ denotes an average pooling layer with a 3×3 kernel, stride 1, and padding 1, ${\text{Conv}}_{1\times 1}\;$ is a pointwise convolutional layer, and the Rearrange operation reshapes tensor $A_{\text{raw}}\in {\mathbb{R}}^{B\times C\times H\times W}$ into $A\in {\mathbb{R}}^{B\times C\times H\times W\times 4}$, where the last dimension corresponds to the four positions within a 2×2 window. Softmax normalization is applied along this final dimension, ensuring that the weights in each window sum to 1.

Standard feature downsampling: a standard downsampling convolution is applied in parallel. As shown in Equation 2.

(2) $$X_{down}=Rearrange\left(f_{ds}\left(X\right)\right)=Rearrange\left(Conv_{3\times 3}^{g,s=2}\left(X\right)\right)$$

The function $f_{ds\;}$ is a 3×3 grouped convolution with stride 2 and group size g = C/16.It expands the number of channels by a factor of 4, producing a tensor of shape ${\mathbb{R}}^{B\times 4C\times H\times W}$. This tensor is then reshaped into $X_{\text{down}}\in {\mathbb{R}}^{B\times C\times H\times W\times 4}$, aligning the expanded four channels with the four pixel values from each input window.

Adaptive weighted fusion: The final output is obtained by element-wise multiplication of the adaptive weights  A with the downsampled feature $X_{\text{down}}$, followed by summing over the four positions. As shown in Equation 3.

(3) $$Y=\sum_{i=1}^{4}A_{:,:,:,:,i}⊙X_{{\text{down}}_{:,:,:,:,i}}$$

Here, ⊙ denotes the Hadamard product (element-wise multiplication). This operation effectively fuses information from each 2×2 window in the input into a single pixel in the output based on content-aware weights.

The LAWDS module provides an efficient and effective mechanism to adaptively preserve the most critical information while reducing the feature map dimensions, making it particularly suitable for tasks requiring fine-grained spatial details.

#### 2.2.2 Lightweight neck design

In the improved network architecture’s Neck section, we replaced the old one-way feature pyramid with a two-way one (BiFPN). This new module learns how to best blend features of different resolutions, making the combination process much more effective (Tan et al., 2020).

**Figure 4:**
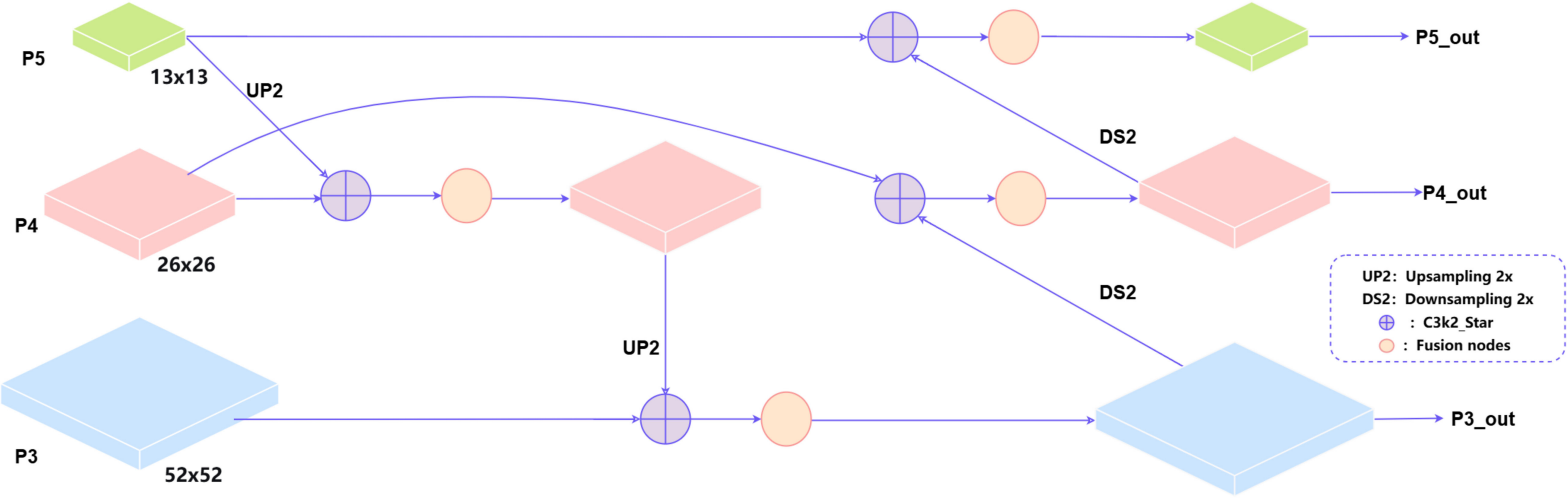
Diagram of the BiFPN structure.

BiFPN achieves multiple cross-scale feature fusions through an ingenious bidirectional structure: first, top-down pathway with upsampling and cross-scale fusion enhances semantic information; then, bottom-up pathway with downsampling and skip connections preserves detailed information. Each final output feature map used for detection simultaneously contains semantic information from higher layers, intrinsic information from the same layer, and detailed information from lower layers. As shown in **Figure 4**.

This module first assigns a trainable parameter vector to each input feature as fusion weights, and uses the ReLU activation function to ensure non-negativity of the weights; to improve numerical stability, a small constant ϵ (1e-4) is added to prevent division by zero errors. Finally, deep fusion of multi-scale features is achieved through weighted summation, generating fused features with rich semantic information. Its core innovation lies in the introduction of a learnable weighting mechanism that can adaptively balance the importance of different input features. As shown in Equation 4.

(4) $$O=\frac{\sum_{i}\;w_{i}\cdot I_{i}}{\sum_{j}\;w_{j}+\in }$$

Here, O denotes the fused output, $I_{i\;}$ represents the input feature maps, $w_{i}$ are the corresponding learnable weights, and ∈ is a small value to prevent numerical instability. In the implementation of this method, ReLU activation function is used to constrain the fusion weights to be non-negative, and normalization is used to make the sum of weights close to 1, thus ensuring the stability and interpretability of the fusion process mathematically. A bidirectional fusion mechanism is established among multiple feature layers in the detection head.

The above fusion shows obvious advantages, Its two-way links between scales enable it to successfully mix high-level meaning and low-level details. The feature representation ability of the model has been significantly enhanced. With the introduction of learnable weight parameters, the model can adaptively adjust the contribution of different feature layers according to the input content. This method also greatly improves the detection accuracy with a very small amount of parameters, and maintains a high computational efficiency. What’s more it enhances robustness to scale distribution variations, particularly improving performance for small object detection.

#### 2.2.3 C3k2_Star module

**Figure 5:**
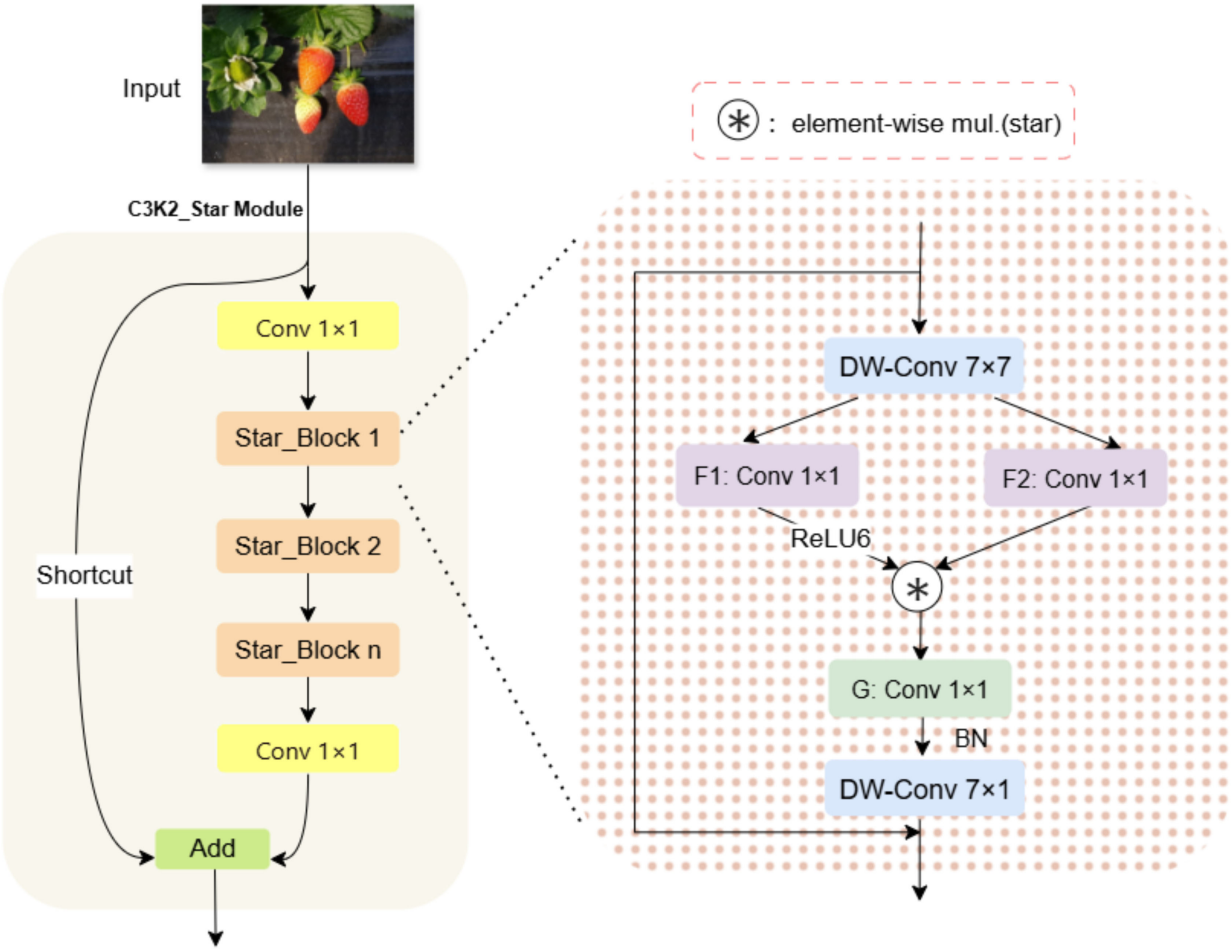
Internal structure diagram of C3k2_Star.

Replace all C3k2 modules with the C3k2_Star module. Compared with the conventional C3k2 module, the C3k2_Star module significantly enhances the representation ability of feature representation by introducing the innovative Star_Block design (Ma et al., 2024). The use of depthwise separable convolutions substantially reduces the number of parameters and computational complexity, while the gated multiplication mechanism enables dynamic feature selection, allowing the network to focus on more informative feature channels. These design choices enable the C3k2_Star module to extract richer and more effective feature representations while maintaining low computational complexity, providing a strong feature foundation for subsequent detection or recognition tasks, and ensuring the overall lightweight and efficiency of the network architecture. As shown in **Figure 5**.

The structure of C3k2_Star module is highly similar to C3k2, and they all follow the design paradigm of split to transform, transform to merger. Given an input feature map $X\in {\mathbb{R}}^{B\times C\times H\times W}$, where B is the batch size, C is the number of channels, and H and W are the spatial height and width, the C3k2_Star module performs feature transformation through its internal structure, producing an output $Y\in {\mathbb{R}}^{B\times C\times H\times W}$.

The core operation of this module is based on the Star_Block structure. Spatial features are extracted from input features by depth separable convolution, and then the extracted features are transformed by two parallel 1×1 convolution layers through the dual-path feature transformation mechanism. These convolutional layers expand the number of channels by a factor of mlp_ratio, resulting in an output shape of ${\mathbb{R}}^{B\times \left(mlp\_ratio\cdot C\right)\times H\times W}$.Then dynamically fuse the dual-path features through a gating mechanism. As shown in Equations 5–8.

(5) $$X^{\prime }={\text{DWConv}}_{7\times 7}\left(X\right)$$

(6) $$X_{1}={ℱ}_{1}\left(X^{\prime }\right)={\text{Conv}}_{1\times 1}\left(X^{\prime }\right)$$

(7) $$X_{2}={ℱ}_{2}\left(X^{\prime }\right)={\text{Conv}}_{1\times 1}\left(X^{\prime }\right)\;$$

(8) $$X_{\text{gated}}=\phi \left(X_{1}\right)⊙X_{2}$$

where ${\text{DWConv}}_{7\times 7}$ denotes a depthwise convolution with a kernel size of 7×7 and the number of groups equal to the input channel count C, enabling efficient spatial feature extraction. ϕ is the ReLU6 activation function, and ⊙ denotes element-wise multiplication. This design enables the model to select important features adaptively.

Subsequently, the gate control output is down-sampled and fused with spatial features, and then the output features are finally generated through residual connection and DropPath regularization. As shown in Equations 9, 10.

(9) $$X_{\text{transformed}}=\text{DWConv}2_{7\times 7}\left(G\left(X_{\text{gated}}\right)\right)$$

(10) $$Y=X+\text{DropPath}\left(X_{\text{transformed}}\right)$$

Here, G is a 1×1 convolutional layer that reduces the number of channels from  mlp_ratio·C back to C, and $\text{DWConv}2_{7\times 7}$ is another 7×7 depthwise convolution used to further enhance spatial features. This design can effectively prevent over-fitting through random depth regularization and ensure the stability of gradient flow. In the C3k2_Star module, multiple Star_Block instances are organized within the C3k2 architecture. The C3k2 architecture first processes the input through two parallel convolutional branches, then concatenates the features from the two branches and passes them through a bottleneck layer. Subsequently, the bottleneck feature is deeply transformed through n cascaded Star_Block instances. As shown in Equations 11–14.

(11) $$X_{\text{branch}1}={\text{Conv}}_{1\times 1}\left(X\right)$$

(12) $$X_{\text{branch}2}={\text{Conv}}_{1\times 1}\left(X\right)$$

(13) $$X_{\text{bottleneck}}=\text{BN}\left(\text{SiLU}\left(\text{Concat}\left(X_{\text{branch}1},X_{\text{branch}2}\right)\right)\right)$$

(14) $$Y={ℳ}_{\text{Star}}^{\left(n\right)}\left(X_{\text{bottleneck}}\right)$$

Here, the ${ℳ}_{\text{Star}}^{\left(n\right)}\;$ represents a sub-network composed of n Star_Blocks.

Through this design, the C3k2_Star module realizes effective feature extraction and transformation, significantly enhancing the model’s representational capacity while maintaining computational efficiency, thereby enabling superior performance in object detection tasks.

#### 2.2.4 LBS-YOLO model network architecture

**Figure 6:**
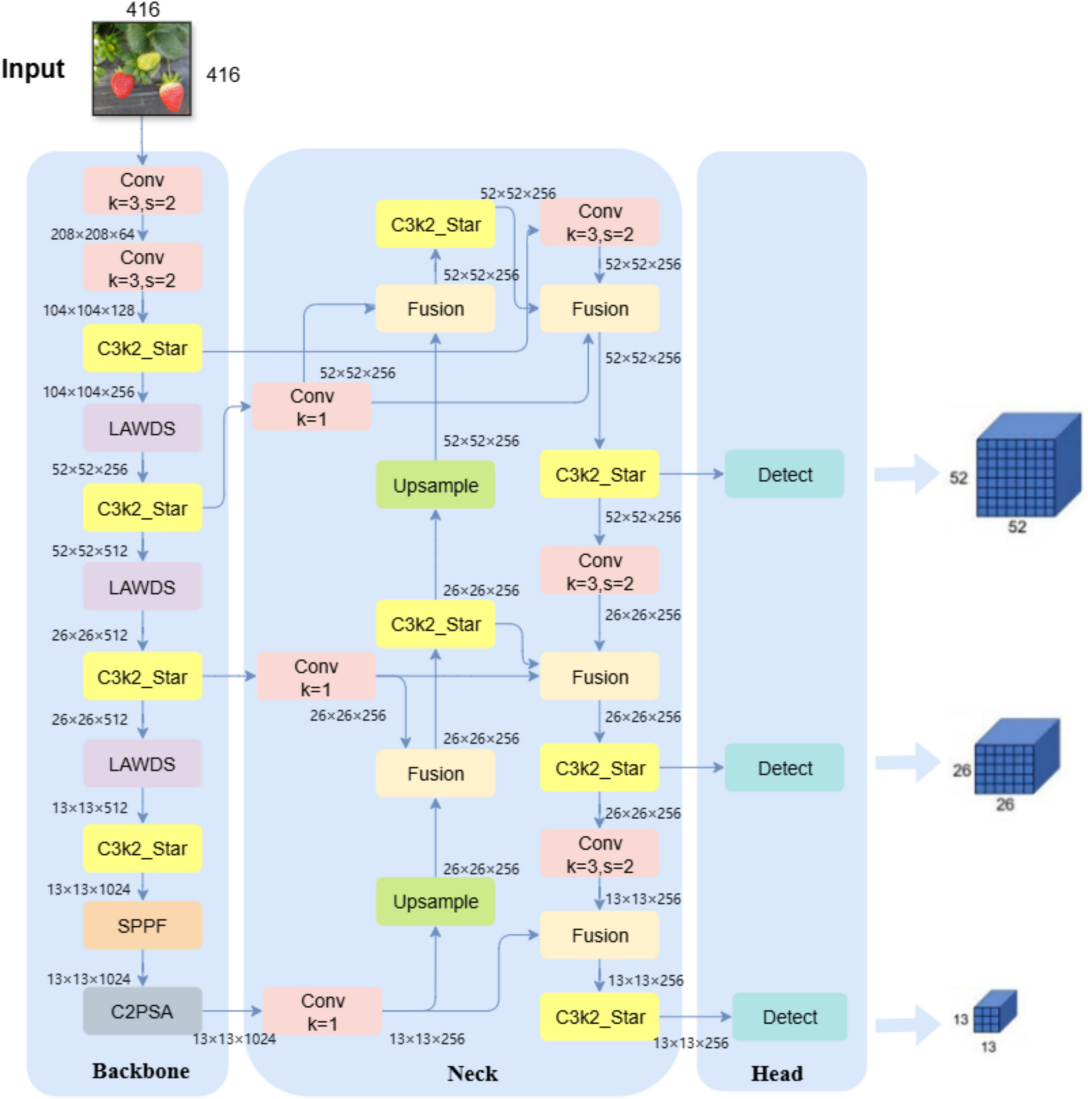
LBS-YOLO overall network architecture diagram.

This study improves upon YOLOv11n and proposes the LBS-YOLO network model, achieving joint optimization of accuracy and efficiency through the introduction of novel modules and fusion mechanisms. First of all, in the backbone network, the original standard convolution downsampling layers at the 3rd, 5th and 7th levels are replaced by LAWDS module, which dynamically fuses features through attention weight, which significantly enhances the feature retention in the downsampling process. Subsequently, all C3k2 modules in the Backbone and Head are upgraded to the C3k2_Star module, leveraging its depthwise separable convolutions and gating mechanism to strengthen feature representation capability, thereby improving the model’s discriminative performance while maintaining lightweight characteristics. Finally, the BiFPN structure is adopted in the feature fusion stage to replace the traditional Concat operation, enabling deeper multi-scale feature fusion through learnable adaptive weights. As shown in the overall network architecture in **Figure 6**.

Test results show that the LBS-YOLO model is more accurate in detection. And significantly enhanced feature fusion efficiency while substantially reducing the number of parameters, providing an optimized solution for lightweight object detection tasks.

## 3 Model training parameters

### 3.1 Experimental environment

The setup of the experiment we used is listed in **Table 1**.

**Table 1:** Model training environment.

Configuration	Parameters
CPU	Intel(R) Xeon(R) Platinum 8255C CPU @ 2.50GHz
GPU	Tesla T4(16GB)
Memory	30GB
System Environment	Linux 5.4.0-166-generic
Programming Environment	Python 3.9.23, Pytorch 2.4.1, CUDA 11.8

### 3.2 Training parameter settings

The training parameters used in this study are set as follows: batch size is set to 8, input image resolution is 416×416, optimizer is SGD, set the initial learning rate to 0.01, and the final learning rate factor to 0.01, momentum size to 0.937, enable mosaic data enhancement throughout the training, train for 150 epochs, and load pre-trained weights to accelerate convergence velocity.

### 3.3 Evaluation metrics

This study uses a range of metrics to evaluate the model. To measure accuracy, it uses Precision (P), Recall (R), F1-Score, and mAP (mAP50, mAP50-95). To measure efficiency, it uses model size, FPS, and FLOPs. Precision (P) measures the proportion of actual positives among predicted positives; recall (R) reflects the proportion of actual positives that are correctly predicted; F1-Score is the harmonic mean of the two. These three metrics evaluate model performance from the dimensions of “prediction accuracy,” “positive class detection rate,” and “balance between the two,” respectively. As shown in Equations 15–17.

(15) $$P=\frac{TP}{TP+FP}$$

(16) $$R=\frac{TP}{TP+FN}$$

(17) $$F1=2\times \frac{P\times R}{P+R}$$

mAP50 is a core evaluation metric for object detection models, used to comprehensively measure model performance, compare different models, and guide optimization directions. Its calculation is based on an Intersection over Union (IoU) threshold of 0.5.

Among these, the intersection ratio (IoU) quantifies the degree of overlap between the predicted boundary box and the real boundary box, which is the basis for judging the correctness of detection. AP50 evaluates the comprehensive performance of a single category under the tradeoff of accuracy and recall. MAP50 extends the evaluation scope to multi-category scenarios, and finally realizes the global evaluation of the overall performance of the model. The specific calculation formula is shown in Equations 18–20.

(18) $$IoU\left(A,B\right)=\frac{A\cap B}{A\cup B}$$

(19) $$AP50=\frac{1}{11}\sum_{r=0.0}^{1.0}\underset{Recall\geq r}{max}Precision$$

(20) $$mAP50=\frac{1}{C}\sum_{c=1}^{C}AP50_{c}$$

where r is the recall node; C is the total number of categories; $AP50_{c}\;$ is the AP50 value for category c.

mAP50–95 calculates the mean of average precision across all categories, where the average precision (AP) for each category is further averaged over the IoU threshold values ranging from 0.5 to 0.95.

The model size is calculated by multiplying the number of parameters by the storage bytes per parameter, measuring the degree of model lightweight design and deployment cost.

FPS measures the number of image frames for a model can process and output per unit time (second), reflecting the model’s inferencing velocity and real-time performance in actual deployment.

FLOPs measure the number of floating-point operations required to execute a single model inference, representing the model’s computational complexity and its requirement for hardware computing capability.

## 4 Model training results analysis

### 4.1 Model training

**Figure 7:**
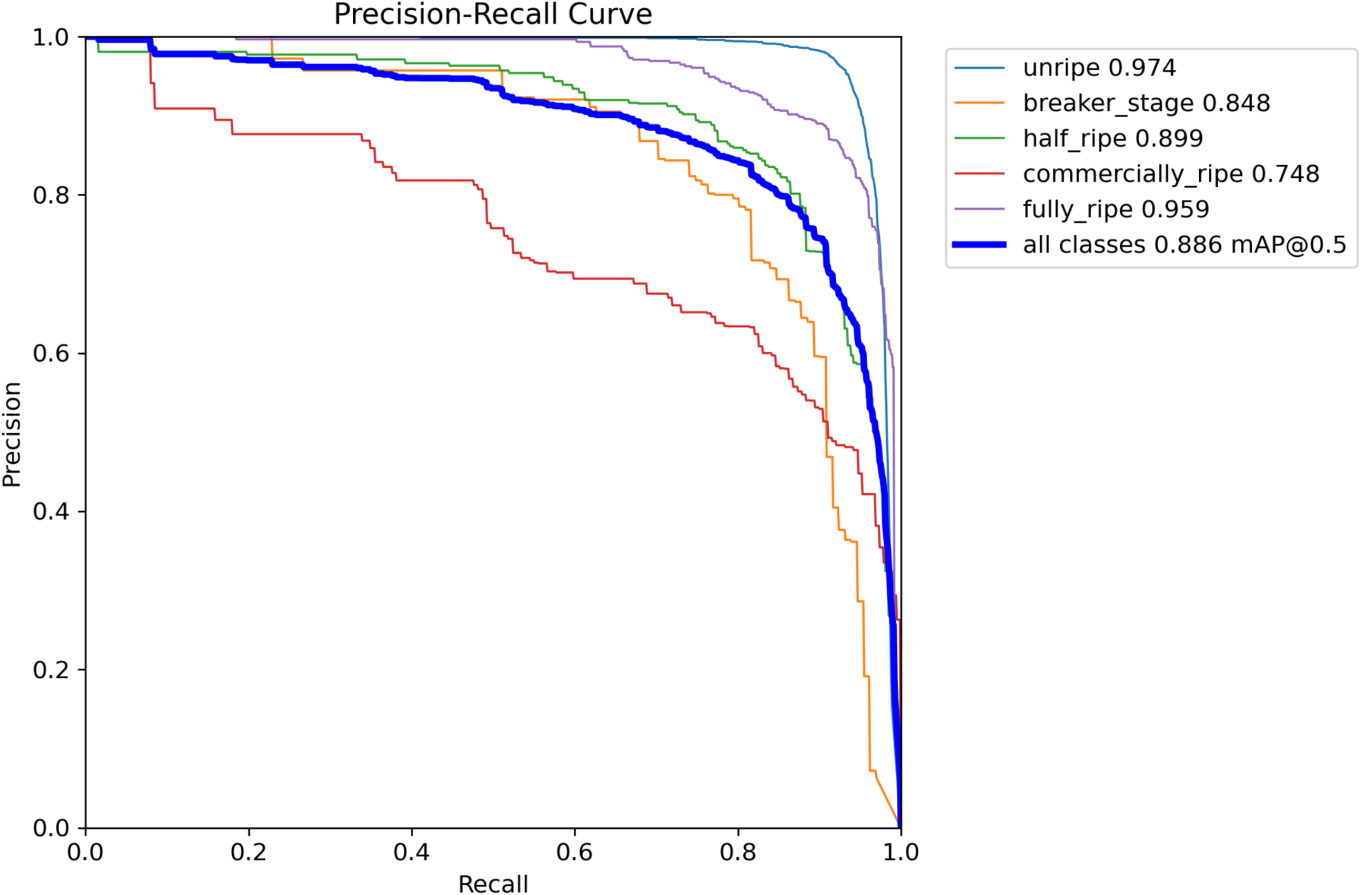
P-R curves of LBS-YOLO.

**Figure 8:**
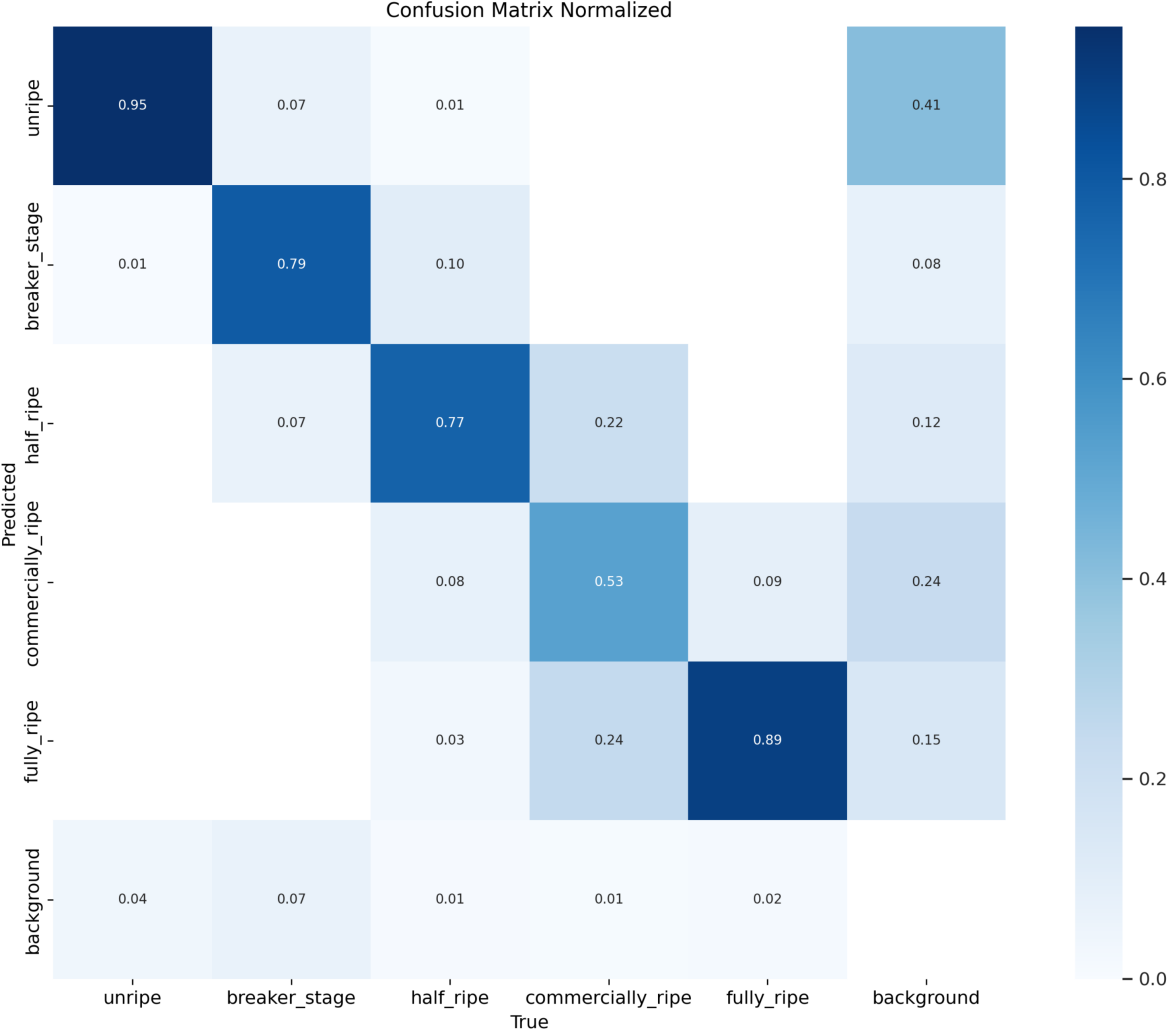
Confusion matrix for LBS-YOLO.

According to **Figures 7**, **8**, the performance of the LBS-YOLO model proposed in this study, as comprehensively assessed through precision-recall (PR) curves and normalized confusion matrices, achieves an average precision (mAP@0.5) of 0.886, demonstrating overall strong classification and detection competency. The unripe category performs relatively standout, with an average precision (AP) reaching 0.974, indicating the model’s effectiveness in distinguishing features of this extreme maturity level. The performance of each category is significantly different. The AP value of commercially-ripe category is the lowest (0.748), and there is a serious mutual misjudgment with half-ripe category. Normalized confusion matrix shows that 22% of half-ripe samples are misjudged as commercially-ripe, while only 53% of commercially-ripe samples are correctly identified and 24% are misjudged as background. This performance difference stems from the high overlap of visual appearance between intermediate maturity categories, and may also be attributed to the unbalanced sample size distribution, which leads to insufficient learning and differentiation of core traits in the model.

To enhance performance, future work will focus on slipstreaming increased sample diversity for intermediate categories, tuning feature extraction modules to strengthen subtle difference discrimination, and verifying the accuracy of background annotations, thereby mitigating category obfuscation and performance variation, and supporting more balanced model performance in maturity level classification tasks.

### 4.2 Ablation experiments

To investigate the specific impact of the three proposed improvement strategies on model detection performance, ablation experiments were conducted by systematically adding different strategy modules. As shown in **Table 2**, Test 1 incorporates only the designed LAWDS module into the baseline model YOLO11n, the LAWDS module achieves intelligent downsampling through a spatial self-adapting weight mechanism, generating region-specific weights via an attention mechanism, which are then normalized by softmax and fused with downsampled features in a weighted manner, enabling dynamic adjustment of the downsampling strategy according to image content, increasing the model mAP50 to 87.7% while reducing the parameter count by 0.34M, indicating that this module enhances feature extraction capability while compressing the model. Test 2 introduces solely the BiFPN structure, the module adopts a learnable weight fusion strategy, using ReLU activation and normalization processing to ensure non-negative weights that sum to 1, thereby self-adapting the balance of contributions from multi-scale traits, significantly improving recall to 87.0% and achieving an mAP50 of 87.9%, with parameters reduced to 1.92M, demonstrating its effectiveness in optimizing multi-scale feature fusion and object coverage. Test 3 incorporates only the C3k2_Star module, the Star_Block and its variants introduce a dual-path gating mechanism, where one path activated by ReLU6 multiplies with another path to enable feature selection and reorganization, combined with depthwise separable convolutions and residual connections to optimize feature extraction, while the CAA attention mechanism further recalibrates feature importance along the channel dimension, achieving the highest precision at 81.8%, highlighting its efficacy in high-confidence detection within complex scenes.

**Table 2:** Ablation experiment results of the LBS-YOLO model.

Model	LAWDS	BiFPN	C3k2_Star	mAP50/%	Precision/%	Recall/%	F1/%	Parameters/M	Weights/MB
YOLO11n				86.4	79.4	83.2	81.2	2.58	5.2
Test 1	✓			87.7	79.7	84.1	81.8	2.24	4.6
Test 2		✓		87.9	78.8	87.0	82.6	1.92	4.0
Test 3			✓	86.7	81.8	81.4	81.6	2.47	5.0
Test 4	✓	✓		88.1	79.1	85.8	82.2	1.63	3.4
LBS-YOLO	✓	✓	✓	88.6	79.8	86.4	82.9	1.60	3.4

Test 4 further integrates LAWDS with BiFPN, the fusion of both components achieves significant effect and synergistic optimization. LAWDS dynamically holds key details in the backbone network through a spatial self-adapting weight mechanism, providing high-quality feature maps for subsequent processing, while BiFPN in the neck network employs a learnable weight fusion strategy to self-adaptively balance multi-scale feature contributions, forming an integrated chain from “intelligent downsampling to optimized feature fusion”, achieving better balance in mAP50, recall rate, and parameter count. Finally, the LBS-YOLO model integrating all strategies achieves optimal comprehensive performance: mAP50 increases to 88.6%, recall rate rises to 86.4%, F1 score reaches 82.9, while model weights are significantly reduced to 3.4MB, indicating a synergistic enhancement effect among modules, collectively accomplishing both improved accuracy and model light-weighting.

### 4.3 Comparative experiments

#### 4.3.1 Model data comparison

To evaluate the performance of the LBS-YOLO model proposed in this study, we conducted a systematic comparison with multiple classic object detection models and current mainstream object detection models, meanwhile we selected the representative YOLOv11n as the baseline model for in-depth analysis. As can be seen from the data in **Table 3**, LBS-YOLO demonstrates significant improvements across multiple core metrics.

**Table 3:** Comparison results of different models.

Model	mAP50/%	mAP50-95/%	Precision/%	Recall/%	F1-Score/%	Weights/MB	Flops/G	FPS
SSD	72.9	61.2	79.5	78.4	78.8	92.6	30.6	55.7
Faster R-CNN	76.9	64.0	73.1	84.2	78.1	158.0	38.9	37.4
EfficientDet	61.1	53.1	76.1	68.4	71.5	142.7	23.3	38.4
RTDETR-l	76.2	64.8	80.2	77.9	79.0	63.1	105.5	60.6
YOLOv5s	85.5	74.2	78.7	83.2	80.9	15.1	6.0	246.2
YOLOv8n	86.1	73.7	78.7	82.7	80.6	5.3	7.0	333.1
YOLOv10n	85.7	74.0	79.8	81.0	80.3	5.4	6.7	394.0
YOLO12n	85.8	73.7	76.7	84.2	80.0	5.2	6.5	271.5
YOLO11n	86.4	74.5	79.4	83.2	81.2	5.2	6.5	320.6
LBS -YOLO	**88.6**	**75.8**	**79.8**	**86.4**	**82.9**	**3.4**	**6.6**	**260.7**

The final bolded line in Table 3 corresponds to our final improved model.

First and foremost, LBS-YOLO achieves 88.6% and 75.8% on the two core metrics mAP50 and mAP50-95, respectively. This outcome not only significantly outperforms the traditional detection model SSD with 72.9% and 61.2%, but also shows a notable boost over Faster R-CNN’s 76.9% and 64.0%. Compared to the baseline model YOLO11n, which achieves 86.4% and 74.5%, LBS-YOLO improves by 2.2 and 1.3 percentage points, respectively, It is particularly noteworthy that the detection accuracy of LBS-YOLO even exceeds that of the large-scale model RTDETR-l, which shows its potential in feature representation. Meanwhile, LBS-YOLO achieves a recall of 86.4%, which is not only higher than YOLO11n’s 83.2%, but also significantly better than SSD’s 78.4% and Faster R-CNN’s 84.2%. Although its precision of 79.8% is slightly lower than RTDETR-l’s 80.2%, its overall F1 score reaches 82.9%, ranking among the top in the compared models and showing good detection reliability.

In terms of computational efficiency, the FLOPs of LBS-YOLO is 6.6G, which is at the same level as the current mainstream lightweight model. Compared with the benchmark model YOLO11n(6.5G), the FLOPS of LBS-YOLO is only slightly increased by 0.1G, which is better than YOLOv8n(7.0G) and YOLOv10n(6.7G). It is significantly ahead of the detection accuracy of YOLOv5s(6.0G), and the amount of calculation is basically the same. Compared with traditional detectors, the computational complexity of LBS-YOLO is only 21.6% of that of SSD and 17.0% of that of Faster R-CNN, and it is much lower than that of heavy-duty architectures such as RTDETR-l(105.5G), which shows its remarkable advantages in computational complexity control. LBS-YOLO’s 260.7 FPS is lower than YOLOv8n’s 333.1 and YOLOv10n’s 394.0, but substantially higher than Faster R-CNN’s 37.4 and RTDETR-l’s 60.6, meeting the requirement for real-time detection. This velocity performance may be the outcome of a trade-off between accuracy and efficiency, achieved by introducing more refined feature processing modules to boost detection performance.

It is worth celebrating that LBS-YOLO has achieved remarkable performance leap while successfully realizing the lightweight of extreme models. Its model size of 3.4MB is not only much smaller than SSD’s 92.6MB and faster R-CNN’s 158.0MB, but also reduced by 34.6% compared with YOLO11n’s 5.2MB, showing excellent parameter efficiency. This shows that LBS-YOLO has greater potential advantages in the use of computing resources, memory consumption, reasoning speed and other aspects. In the future, it can run on edge devices that have limited computing power, like mobile phones or embedded systems. Experimental results indicate that compared to mainstream lightweight models, LBS-YOLO demonstrates advantages in detection accuracy and achieves an effective balance between model efficiency and performance, showing good application potential.

#### 4.3.2 Model visualization comparison

**Figure 9:**
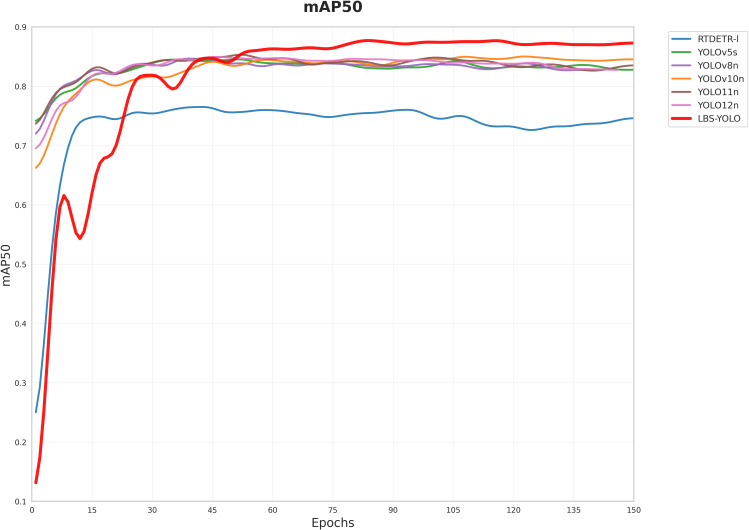
Comparison of mAP50 across various models.

The mAP50 training curves of YOLO series model and LBS-YOLO model are put in a picture. As shown in **Figure 9**, the mAP50 curves of all models conform to the training mode of the target detection model, showing a trend of steady rise, late convergence and gentle fluctuation. This indicates that the experimental hyperparameter settings, dataset distribution, and other conditions are stable, eliminating biases caused by abnormal experimental conditions and laying the foundation for comparative analysis.

The LBS–YOLO curve exhibits significant fluctuations initially, which is a normal behavior during model adaptation following architectural innovation. All models are trained based on pretrained weights. The LBS–YOLO model reconstructs the feature fusion module and adjusts network depth based on YOLO11, leading to mismatches between original feature maps and the new architecture. These fluctuations can obviously reflect the process that the model inherits the pre-training knowledge and adapts to the new structure, which shows that the pre-training weight provides effective guidance for the new model.

As shown in **Figure 9**, LBS-YOLO quickly converges after a short adaptation at the initial stage of training, and finally its performance is stable beyond the baseline model. This improvement is due to the optimization of redundant feature processing and the introduction of dynamic anchor point adjustment mechanism. While retaining the advantages of YOLOv11n core architecture, the improved structure significantly enhances the model’s adaptability to complex scenarios while maintaining its original advantages. This fully demonstrates the effectiveness of the structural improvements.

**Figure 10:**
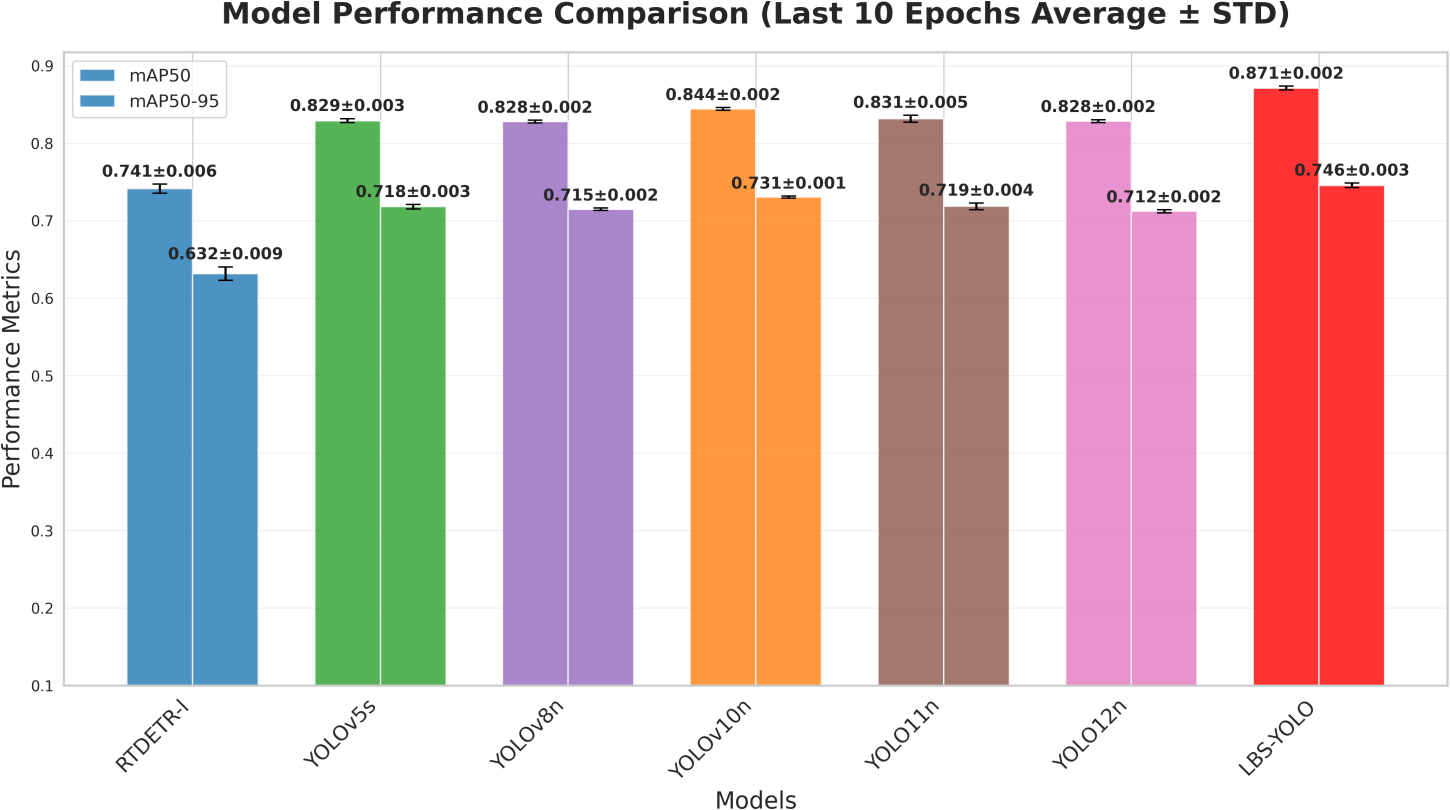
Average ± std. dev. of the last 10 epochs.

As shown in **Figure 10**, he proposed LBS-YOLO model in this study demonstrates outstanding comprehensive performance and stability in the average performance comparison over the last 10 runs. The model achieves the highest weighted average score, indicating optimal overall performance. Meanwhile, it exhibits the smallest standard deviation, confirming high output reliability and robustness. All models achieve 100% baseline accuracy and value ratio, while the proposed model further advances in more refined weighted metrics, significantly surpassing the comparative baselines, thereby fully validating the effectiveness and advancement of the model improvements.

### 4.4 Detection performance comparison

#### 4.4.1 Comparative analysis of different model detection

**Figure 11:**
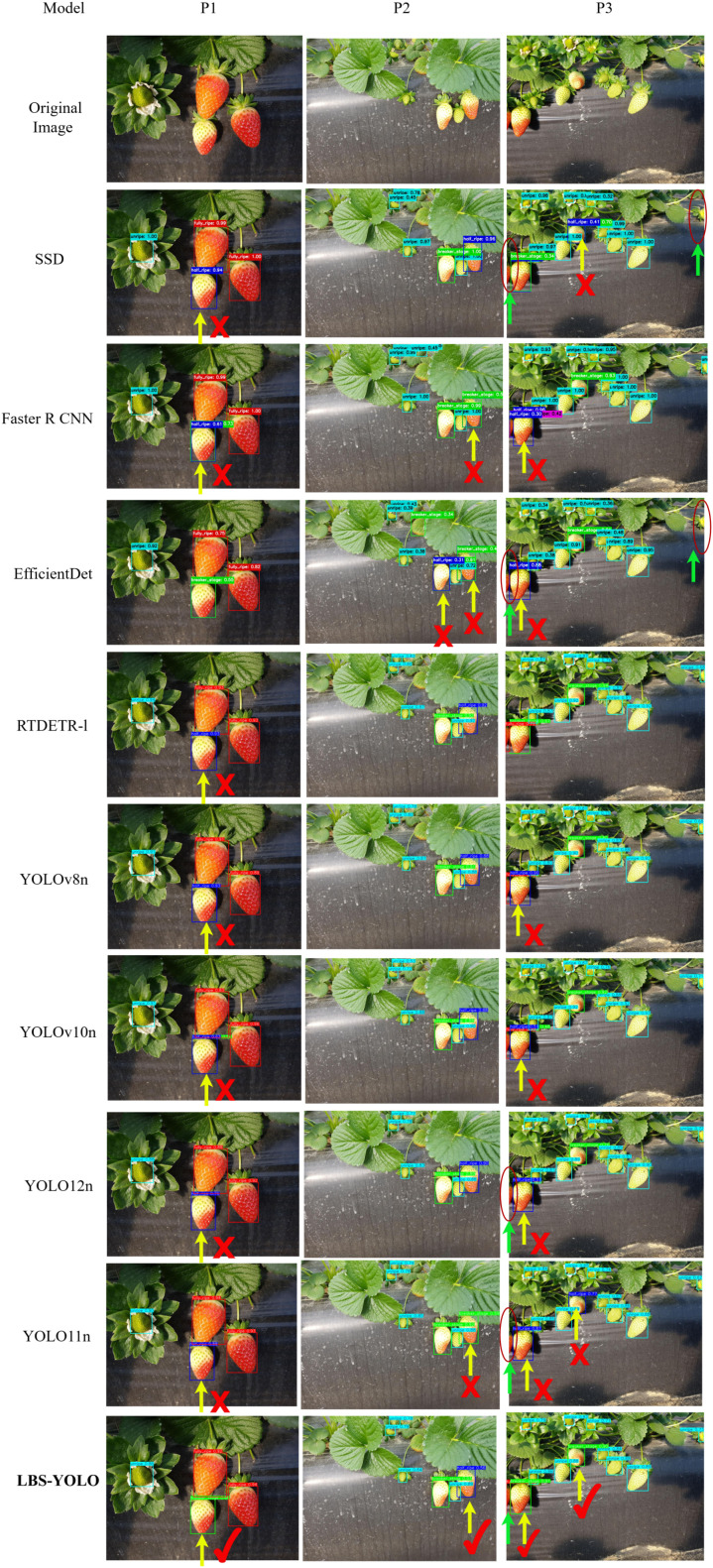
Comparison of detection performance across different models.

As shown in the comparison of detection results in **Figure 11**, From the detection outcomes in three scenes—P1, a relatively ideal scene; P2, a scene with small-sized strawberries; and P3, a scene with edge-positioned strawberries—LBS-YOLO shows certain variations in performance compared to the other eight models, and overall demonstrates superior detection stability. To facilitate observation and differentiation, the graph uses yellow arrows and “×” marks to call out strawberries that are falsely detected, and yellow arrows and “√” marks” to indicate correct detections. LBS-YOLO exhibits no false positive callouts or repetition callouts across all three scenes, providing relatively higher assurance of detection accuracy. In P1, only EfficientDet and LBS-YOLO basically achieve fully correct category decisions, while the other seven models incorrectly classify one strawberry that has not yet reached semi-ripeness as semi-ripe. In scene P2, most models such as YOLOv10n, YOLO12n, and RTDETR-1 achieve good detection performance, but false detections are clearly observed in YOLO11n, EfficientDet, and Faster R-CNN. In scene P3, all models except RTDETR-l and LBS-YOLO exhibit false detection cases. Additionally, during observation, one instance of repeated annotation is found in both SSD and Faster R-CNN.

Regarding missed detections, green arrows combined with dark red circles are used in the figures for callouts. No missed detections are discovered in LBS-YOLO across all three scenes, demonstrating comprehensive grabbing of visible strawberries, small-sized strawberries, and edge-positioned strawberries. In P3, SSD, EfficientDet, YOLO12n, and YOLO11n miss the edge strawberry located at the far left of the picture, while SSD and EfficientDet also fail to detect the edge strawberry on the far right. In terms of localization accuracy, LBS-YOLO shows high alignment between its detection bounding boxes and fruit outlines. In P1, leaf inclusion is minimal; in P2, tight fitting around small strawberries is achieved with less redundant spatial area; in P3, better adaptation to irregular edge strawberry shapes is demonstrated.

Other models exhibit varying degrees of bounding box fitting issues: the bounding boxes of YOLOv10n, SSD, Faster R-CNN, YOLO12n, and EfficientDet are slightly larger, potentially including a small number of leaves in P1, possibly providing incomplete coverage for small strawberries in P2, and showing overlapping boxes at repeated callouts in P3; the box tightness of YOLO11n and RTDETR-1 is at a medium level, with potential edge cropping on small strawberries in P2 and possible top cropping on some strawberries in P3; YOLOv8n suffers from redundant or drifted bounding boxes.

Generally speaking, in the three scenarios, LBS-YOLO model shows a balanced detection ability and maintains a stable performance. The model effectively avoids false detection and missed detection, and the detection frame is highly consistent with the actual fruit contour, which reflects its superior attribute in target detection accuracy.

#### 4.4.2 Heatmap comparison and parse in occlusion scenes

In this section, GradCAM heatmap is used to study the feature learning behavior of LBS-YOLO in strawberry detection task, and its applicability under the condition of partial occlusion caused by leaves or fruits is evaluated, and its performance is compared with that of baseline YOLO11n.

**Figure 12:**
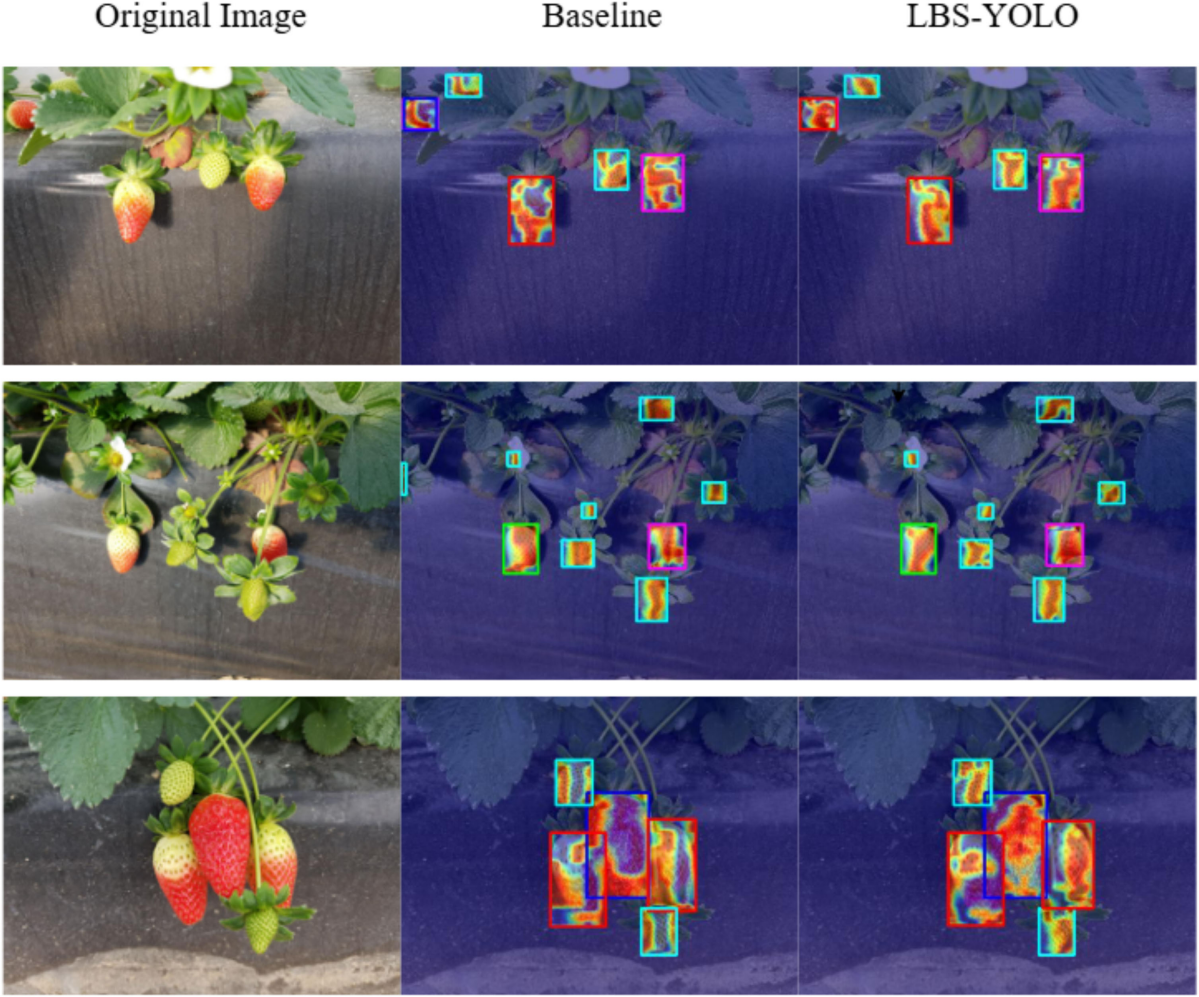
Comparison of heatmap effects between baseline and LBS-YOLO.

As shown in **Figure 12**, the baseline model has problems with scattered attention or blurry focus in its heatmap. In areas where strawberries overlap with leaves and stems, the model fails to focus accurately on the strawberries themselves. Instead, it includes some background textures in important areas, which shows that its ability to tell different objects apart is limited. In contrast, the heatmap generated by LBS-YOLO is more compact, almost completely covering the strawberry body while showing minimal attention to irrelevant background areas, demonstrating superior target semantics grabbing capability.

The baseline model’s heatmap often misses the real edges of strawberries, with heat spilling out or not reaching the actual border, causing inaccurate placement. On the other hand, LBS-YOLO’s heatmap fits perfectly to the strawberry’s shape. It works well for both complete strawberries and those that are partly blocked, giving a more precise target location. For small target detection capability, due to the presence of a large number of immature small strawberries in the strawberry growth scene, the baseline either shows insufficient attention with bluish color on small strawberries or confuses them with the surrounding background, resulting in low distinguishability.

In contrast, LBS-YOLO can generate a clear and high-brightness region of interest even for small strawberries, which forms a very high contrast with the surrounding background, thus showing excellent small target detection performance. Taking all points into account, LBS-YOLO performs much better than the baseline model in grabbing target meaning, finding object edges, and detecting small objects.

## 5 Discussion

This study has systematically improved upon existing issues such as non-standard maturity grading, difficulties in supporting refined requirements like graded harvesting, frequent misdetection and missed detection due to obstruction by branches, leaves, and fruits, and the challenge of balancing accuracy and efficiency.

In the model structure, the LAWDS, C3k2_Star, and BiFPN parts work together as a whole. The LAWDS part performs smart down-sampling and keeps the important details by adjusting weights across space. The C3k2_Star part greatly improves the model’s ability to spot small differences in ripeness by using depthwise separable convolutions and a gating system. The BiFPN part makes multi-scale feature fusion more efficient by learning how to best weigh different inputs. With these improvements, the mAP@0.5 of LBS-YOLO reached 88.6%, the recall rate reached 86.4% and the F1 score reached 82.9%. The storage occupation is only 3.4MB, and the parameter amount is 1.6 million, which is better than YOLO11n.

However, the study also exposes some problems worthy of deeper thinking. The significant confusion between the semi-ripe and commercially-ripe categories (the latter with an AP of only 0.748) reveals the inherent challenges in fine-grained classification. This phenomenon may be due to the inherent high overlap of the visual characteristics of the two categories and the lack of balance in data distribution, which interferes with model learning. Moreover, although the model’s FPS (260.7) meets the basic requirements for real-time detection, a performance gap remains when compared to state-of-the-art lightweight models such as YOLOv10n, reflecting the intrinsic trade-off between accuracy and velocity.

Future studies should focus on constructing practical and complex strawberry data sets, validating model robustness in broader and more diverse real-world scenes, while exploring more effective feature disentanglement methods to address category obfuscation in fine-grained taxonomy, and actively improving detection velocity to enhance deployment efficiency on hardware.

## 6 Conclusion

This study systematically improved the YOLOv11n model to address the practical problem of balancing detection accuracy and efficiency in lightweight models during strawberry intelligent harvesting, proposing a lightweight object detection model named LBS-YOLO. The main conclusions of this study are as follows:

1. For the first time, a strawberry maturity dataset containing five fine-grained grades was constructed according to the agricultural industry standard (NY/T 2787-2015), enabling the model output to directly interface with grading harvesting operations and enhancing its practical value in real-world strawberry harvesting scenes.
2. By introducing a lightweight self-adapting weighted downsampling module (LAWDS) into the backbone network of YOLOv11n, further integrating the bidirectional feature pyramid network (BiFPN) structure, and completely replacing the original C3k2 blocks with C3k2_Star modules, the feature discrimination capability was enhanced through gating mechanisms and depthwise separable convolutions. Finally, the fully integrated LBS-YOLO model achieved an mAP50 of 88.6%, a recall of 86.4%, and an F1 score of 82.9, with a model weight of only 3.4 MB, significantly outperforming the raw model and achieving a better balance between detection accuracy and model lightweighting.
3. The explainability of the modules proposed in this study and their generalization capability on other detection tasks still require further exploration. Future research will be dedicated to further refinement of the module architecture, along with the development of datasets tailored to specific complex local scenes, attempting to extend the app extension to multiclass and small-target detection tasks, and exploring dynamic inferencing mechanisms and hardware-aware collaborative optimization to enhance the practicality and adaptability of the algorithm in real-world scenes.

This series of optimizations affords more robust technical support for the digital administration and intelligent harvesting of strawberry growth procedures. future studies could further expand application scenes and deepen the model’s adaptability to complex light environments. at the same time, exploring deeper fusion between detection outcomes and production decision-making can more directly transform technical advantages into industrial benefits.

## Data Availability

The original contributions presented in the study are included in the article/supplementary material. Further inquiries can be directed to the corresponding author.

## References

[B1] Adams S. A. Paparozzi E. T. Pekarek R. Lambe D. P. Meyer G. Conley M. E. . (2021). University research on winter growing of container-grown strawberries translates to grower’s farm trial. Int. J. Fruit Sci. 21, 1104–1113. doi: 10.1080/15538362.2021.1994510

[B2] Balaji A. Harini T. Kavitha R. Pavithra S. (2025). “ Performance analysis of YOLO models for strawberry ripeness detection in challenging environments,” in 2025 International Conference on Data Science, Agents & Artificial Intelligence (ICDSAAI), Chennai, India. 1–5 (IEEE). doi: 10.1109/ICDSAAI65575.2025.11011833

[B3] Charoenwoodhipong P. Zuelch M. L. Keen C. L. Hackman R. M. Holt R. R. (2025). Strawberry (Fragaria x Ananassa) intake on human health and disease outcomes: a comprehensive literature review. Crit. Rev. Food Sci. Nutr. 65, 4884–4914. doi: 10.1080/10408398.2024.2398634, PMID: 39262175

[B4] Gao S. Cui G. Wang Q. (2025). WCS-YOLOv8s: an improved YOLOv8s model for target identification and localization throughout the strawberry growth process. Front. Plant Sci. 16. doi: 10.3389/fpls.2025.1579335, PMID: 40718025 PMC12289670

[B5] Guo J. Yang Z. Karkee M. Jiang Q. Feng X. He Y. (2024). Technology progress in mechanical harvest of fresh market strawberries. Comput. Electron. Agric. 226, 109468. doi: 10.1016/j.compag.2024.109468

[B6] He L. Wu D. Zheng X. Xu F. Lin S. Wang S. . (2025). RLK-YOLOv8: multi-stage detection of strawberry fruits throughout the full growth cycle in greenhouses based on large kernel convolutions and improved YOLOv8. Front. Plant Sci. 16. doi: 10.3389/fpls.2025.1552553, PMID: 40201777 PMC11975920

[B7] Hernández-Martínez N. R. Blanchard C. Wells D. Salazar-Gutiérrez M. R. (2023). Current state and future perspectives of commercial strawberry production: A review. Scientia Hortic. 312, 111893. doi: 10.1016/j.scienta.2023.111893

[B8] Hodgdon E. A. Conner D. S. McDermott L. G. Pritts M. P. Handley D. T. Orde K. M. . (2024). A current view on strawberry production practices and trends in the northeastern United States and Canada. HortTechnology 34, 574–584. doi: 10.21273/HORTTECH05457-24

[B9] Li Z. Wang J. Gao G. Lei Y. Zhao C. Wang Y. . (2024). SGSNet: a lightweight deep learning model for strawberry growth stage detection. Front. Plant Sci. 15. doi: 10.3389/fpls.2024.1491706, PMID: 39717733 PMC11664550

[B10] Li J. Zhu Z. Liu H. Su Y. Deng L. (2023). Strawberry R-CNN: Recognition and counting model of strawberry based on improved faster R-CNN. Ecol. Inf. 77, 102210. doi: 10.1016/j.ecoinf.2023.102210

[B11] Liu J. Guo J. Zhang S. (2025). YOLOv11-HRS: an improved model for strawberry ripeness detection. Agronomy 15, 1026. doi: 10.3390/agronomy15051026

[B12] Ma X. Dai X. Bai Y. Wang Y. Fu Y. (2024). Rewrite the stars. 2024 IEEE/CVF Conference on Computer Vision and Pattern Recognition (CVPR), 5694–5703. doi: 10.48550/ARXIV.2403.19967

[B13] Ma H. Zhao Q. Zhang R. Hao C. Dong W. Zhang X. . (2025). YOLOv11-GSF: an optimized deep learning model for strawberry ripeness detection in agriculture. Front. Plant Sci. 16. doi: 10.3389/fpls.2025.1584669, PMID: 40909907 PMC12405203

[B14] Newerli-Guz J. Śmiechowska M. Drzewiecka A. Tylingo R. (2023). Bioactive Ingredients with Health-Promoting Properties of Strawberry Fruit (Fragaria x ananassa Duchesne). Molecules 28, 2711. doi: 10.3390/molecules28062711, PMID: 36985683 PMC10059084

[B15] Pinochet D. Agehara S. (2024). Early-season yield fluctuations of strawberry (Fragaria ×ananassa Duch.) follow a bimodal Gaussian model. Scientia Hortic. 336, 113440. doi: 10.1016/j.scienta.2024.113440

[B16] Rother C. Kolmogorov V. Blake A. (2004). GrabCut”: interactive foreground extraction using iterated graph cuts. ACM Trans. Graph 23, 309–314. doi: 10.1145/1015706.1015720

[B17] Tan M. Pang R. Le Q. V. (2020). “ EfficientDet: scalable and efficient object detection,” in 2020 IEEE/CVF Conference on Computer Vision and Pattern Recognition (CVPR), Seattle, WA, USA. 10778–10787 (IEEE). doi: 10.1109/CVPR42600.2020.01079

[B18] Tao Z. Li K. Rao Y. Li W. Zhu J. (2024). Strawberry maturity recognition based on improved YOLOv5. Agronomy 14, 460. doi: 10.3390/agronomy14030460

[B19] Tenea G. N. Reyes P. (2024). Bacterial community changes in strawberry fruits (Fragaria × ananassa variety “Monterey”) from farm field to retail market stands, an indicator of postharvest contamination. Front. Microbiol. 15. doi: 10.3389/fmicb.2024.1348316, PMID: 38435684 PMC10904649

[B20] Wang C. Wang H. Han Q. Zhang Z. Kong D. Zou X. (2024). Strawberry detection and ripeness classification using YOLOv8+ Model and image processing method. Agriculture 14, 751. doi: 10.3390/agriculture14050751

[B21] Yang S. Wang W. Gao S. Deng Z. (2023). Strawberry ripeness detection based on YOLOv8 algorithm fused with LW-Swin Transformer. Comput. Electron. Agric. 215, 108360. doi: 10.1016/j.compag.2023.108360

[B22] Yu H. Chen H. Fang X. Mu H. Zhou Y. Tao F. . (2015). Technical specification for harvest,storage and transportation of strawberries. Available online at: https://std.samr.gov.cn/hb/search/stdHBDetailed?id=AF6B1A28B32F010FE05397BE0A0A83CE (Accessed March 20, 2025).

[B23] Yu H. Qian C. Chen Z. Chen J. Zhao Y. (2025). Ripe-detection: A lightweight method for strawberry ripeness detection. Agronomy 15, 1645. doi: 10.3390/agronomy15071645

[B24] Zeneli F. Ventura V. Frisio D. G. (2024). Sustainable fresh strawberry consumption: environmental, genetically modified food, and climate concerns in Europe and North Africa. Front. Sustain. Food Syst. 8. doi: 10.3389/fsufs.2024.1442074

[B25] Zhang X. Li H. Li S. Zhang W. Xian Z. (2022). Rapid detection and classification of strawberries based on EfficientDet-D1. J. Huazhong Agric. Univ. 41, 262–269. doi: 10.13300/j.cnki.hnlkxb.2022.06.031

[B26] Zhou C. Hu J. Xu Z. Yue J. Ye H. Yang G. (2020). A novel greenhouse-based system for the detection and plumpness assessment of strawberry using an improved deep learning technique. Front. Plant Sci. 11. doi: 10.3389/fpls.2020.00559, PMID: 32582225 PMC7283502

